# Sleep, Stress, and Recovery as Predictors of Injury Risk in Soccer Players: A Systematic Review

**DOI:** 10.3390/healthcare14020236

**Published:** 2026-01-17

**Authors:** Enrique Cantón, Joel Raga, David Peris-Delcampo

**Affiliations:** 1 Department of Basic Psychology, Faculty of Psychology, Universitat de València, 46010 Valencia, Spain; enrique.canton@uv.es; 2 Col·legi Oficial de Psicologia de la Comunitat Valenciana—COPCV, 46003 Valencia, Spain; jragamartinez@gmail.com; 3 Department of Psychological, Personality, Evaluation and Treatment, Faculty of Psychology, Universitat de València, 46010 Valencia, Spain

**Keywords:** soccer, injury risk, football players, melatonin, sleep

## Abstract

**Introduction**. Sleep is an essential component in the recovery, performance, and injury prevention processes of soccer players. Associated psychological variables, such as the balance between stress and recovery, have been less explored, despite their potential influence on rest and injury vulnerability. This study aims to examine the relationship between sleep quality, quantity, and chronotype and injury risk in soccer players, also incorporating the modulating role of stress and recovery. **Method**. A PRISMA systematic review was conducted using searches in ScienceDirect, PubMed, Ovid, EBSCO, MDPI, Springer Nature Link, SPORTDiscuss (full text), and Dialnet. Original studies and reviews on sleep and its relationship with sports injuries in soccer players or comparable athletic populations were included. Eighteen studies were selected that addressed sleep indicators (quality, quantity, chronotype), injury incidence, and, to a lesser extent, measures of stress and recovery using instruments such as the RESTQ-Sport or wellness questionnaires. **Results**. There is evidence of an association between poor sleep quality or quantity and an increased risk of injury or illness. Chronotype is an emerging variable of interest, although still insufficiently researched. Regarding stress and recovery, direct evidence is limited, although studies that address this issue show that an imbalance between these two dimensions negatively impacts sleep quality and increases susceptibility to injury. **Conclusions**: Sleep and the stress–recovery balance are key and interdependent factors in the risk of injury in soccer players. Future research should consider including these variables to further understand the mechanisms underlying the injury process and optimize prevention and recovery strategies.

## 1. Introduction

Football or soccer is a team sport played on a grass or artificial pitch. Two teams of 11 players each compete with the objective of scoring by putting the ball into the opposing team’s goal to win the match.

This sport began in 1863 in the United Kingdom (Great Britain and Ireland), where the first international competition was held. Over the decades, football has become and remains the most popular sport in the world [1], with a participation of more than 265 million people [2].

Since the end of the last century and the beginning of this one, football has evolved significantly in both its dynamics and its organizational structure. Previously, football clubs did not have the same roles and positions as they do today; in fact, the head coach was often the only one present. However, nowadays, the coaching staff of any club includes a wider range of professionals, including head and assistant coaches, fitness coaches, physiotherapists, doctors, analysts, technical assistants, and fortunately, in some cases, a sports psychologist as well.

Furthermore, the game itself has also evolved. This sport now demands much more than in the past, with greater physical, technical, and tactical requirements. Moreover, football requires some activities such as sprinting, changing direction, jumping, grabbing the ball, as well as technical skills, for example, dribbling, passing, shooting the ball [3].

In particular, emotional and psychological aspects have acquired a fundamental role in performance.

However, it is clear that football does not evolve on its own. Various factors have influenced this process: technological, economic, social, emotional, and competitive. All of these have contributed to the way the “king of sports” is perceived, and to the fact that those involved in the game must (in most cases) be fully prepared to meet these new demands.

Being a football player is not the same as being a footballer. The two terms may seem synonymous, but they are not. Being a football player encompasses all those who practice the sport, whether professionally, as an amateur, or simply as a hobby. However, being a footballer goes much further. Footballers are those who practice the sport professionally; that is, the sport is their profession and their job. They have a formal contract with a club that has a serious and disciplined structure. It is crucial to emphasize that all footballers are football players, but not all football players are footballers. With regard to the aforementioned factors, it is important to consider everything they are constantly subjected to. Footballers, in addition to being athletes, are also people, physically and psychologically prepared to compete daily. However, they are increasingly exposed to greater physical and psychological pressures, which can be detrimental to their well-being. It is in this context that stress often arises, since if these demands are not adequately compensated for through recovery, it generates accumulated fatigue, impaired performance, and greater physical and psychological vulnerability. Some of the factors that can negatively affect football players are intense matches (high intensity and number of training sessions/matches), travel, unfamiliar environments for sleeping, nighttime schedules that disrupt circadian rhythms, and a short recovery phase [4].

Furthermore, several studies have shown that elite athletes need more than 72 h to recover from muscle damage and inflammation in order to return to peak physical performance levels [3].

Therefore, recovery refers to the restoration of these physiological and mental resources after exertion. Regarding this concept, there are two terms: incomplete or inadequate recovery, and excessive recovery [3].

On the one hand, incomplete or inadequate recovery can lead to stress accumulation, maladaptation, and decreased performance [5]. This accumulation of stress, whether physical or emotional, can disrupt sleep patterns, reduce the perception of fatigue, and increase a player’s vulnerability to injury [4], thus affecting their recovery capacity [6]. This is where fatigue comes into play.

Fatigue can be defined as a decrease in performance associated with muscle activity [3]. Fatigue can be divided into two facets: acute and chronic. Acute fatigue occurs after a single match and is characterized by declines in physical performance that develop over days and hours [3]. In contrast, chronic fatigue is characterized by having a longer duration and being associated with major causes, being a symptom of other diseases. Similarly, there is another type of fatigue that is complementary to physical activity, which causes cognitive exhaustion—mental fatigue.

Mental fatigue occurs when a football player is exposed to numerous and demanding environmental factors, especially during competition. Football matches can cause physical stress in athletes, as well as psychological stress, due to the need to concentrate for extended periods, the need to maintain a high level of perception, and the difficulty of making decisions under pressure from opponents [3]. This is due to the prolonged duration to which they have to be fully committed.

For this reason, some athletes, even when physically fatigued, can continue performing automated actions, albeit with less intensity. However, their performance will show a significant decrease. This means that mental fatigue also impacts physical performance.

On the other hand, excessive recovery can lead to inappropriate stress, inadequate adaptability, and minimal performance improvement [5].

In short, besides the role of sleep as another important factor in injury prevention, recent scientific literature points to the need to consider other related variables, such as the stress–recovery balance. This pairing influences both sleep quality and the body’s ability to repair physiological damage resulting from exertion. Therefore, it has been shown that a prolonged imbalance between demand and recovery significantly increases the risk of injury in a soccer player, because in professional soccer, the risk of injury is high [4].

With all of the information mentioned above, the concept of injury is defined as any event that requires medical intervention by an athletic trainer and results in the complete restriction of one or more practices or games [7]. To this definition, we must add the aspect of mental health in athletes, since it also requires medical intervention and the possibility of being sidelined for the time necessary for recovery.

Based on this definition, classified injuries into five types: 1. Mild: requires treatment, but can continue playing with a bandage; 2. Moderate: requires treatment and modification of training, loads, etc.; 3. Severe: requires treatment, sometimes surgical and prolonged inactivity; 4. Severe with chronic deterioration, which prevent a return to previous performance; 5. Severe with permanent disability [8].

Given this classification, many soccer players suffer injuries throughout the season. Specifically, one study mentions an average of 5.63 injuries per 1000 h of exposure in female soccer players. This is a lower incidence of injuries than the 8.1/1000 h in male soccer. However, injuries are more severe in female players, particularly anterior cruciate ligament (ACL) and ankle syndesmosis injuries [1]. This may be primarily due to the fact that women are not subjected to the same number of matches as men in terms of competitions. Many elite female footballers have suffered serious injuries requiring surgical treatment over the past two years.

Consequently, injuries have numerous repercussions for athletes. Suffering an injury causes physical and psychological changes, limits their ability to participate in any type of extracurricular activity (in the case of severe injuries), causes changes at the family and social level, and, of course, rehabilitation requires time and effort. Furthermore, it is crucial to consider the psychological impact on the player, taking into account external factors, their specific characteristics, and their coping skills.

**Figure 1:**
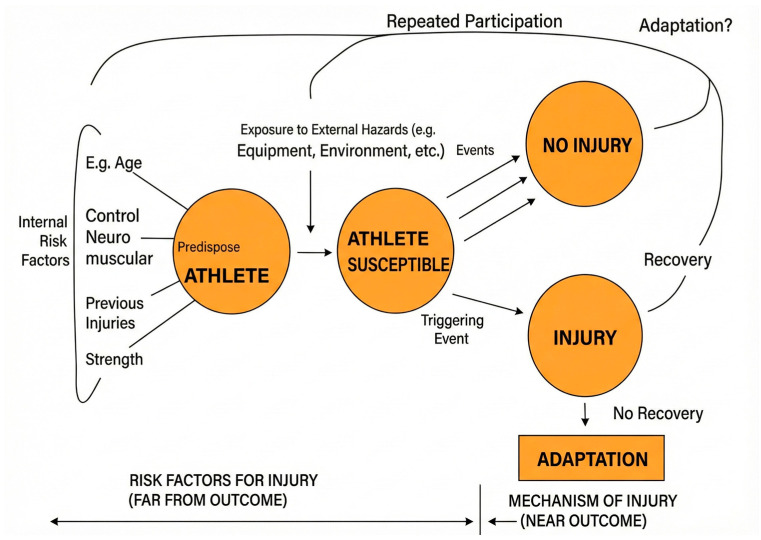
The Meuwisse model.

The Meuwisse Model in 2007 (Figure 1: Meuwisse Model) justifies to some extent the possible causes of suffering injuries.

The model assumes that intrinsic factors can be additive or potentiated, and in turn, interact with extrinsic factors to produce a combined effect, thereby modifying the subject’s predisposition and susceptibility to injury. However, the participant’s risks are dynamic and can change constantly.

Since this cyclical model, of interaction between events and factors, is considered, it is repeated periodically and the final results will depend on the magnitude of the inciting events and their interaction with other factors, both internal and external. In addition, the model has three phases:

1. Predisposition: the descriptive variables of the athlete; age, injury history, rest, fatigue …
2. Susceptibility: This phase consists of the transition to susceptibility, where it interacts with external factors: infrastructure, sport, environment …
3. Injury/No injury: In this phase comes the interaction between extrinsic and intrinsic factors where the probability of suffering injuries or not occurs.

Therefore, given the importance of injuries in the world of football today and all the likely factors that influence them, the objective of this study is to focus on the impact of sleep (quality, quantity, chronotype, etc.) on injury risk in footballers, as well as to explore the role of related variables such as the stress–recovery balance. Including this latter dimension is fundamental to understanding the injury phenomenon from a more comprehensive and biopsychosocial perspective.

As previously mentioned, fatigue leads to muscle loss and damage in the body. The best way to remedy this is through rest and recovery via sleep. Numerous studies state that athletes should sleep at least 7 h a day [9], healthy athletes should sleep between 7 and 9 h each night [5] and that a sustained imbalance between stress and recovery (subjective perception of fatigue) is related to a higher incidence of injuries, with a directly positive relationship [4]. Therefore, sleep is the best way to prevent physical and mental deterioration, as well as to reduce stress, since players are continuously exposed to high intensity and stress.

However, there is growing evidence that dysfunctional sleep patterns negatively affect performance, physical health, and psychological well-being. This may be because sleep deficits cause physiological and hormonal changes [10], including increased cytokine secretion, attenuated antibodies, and alterations in the central nervous system and the immune system. Furthermore, the psychological impact is even greater because sleep disturbance is linked to altered emotional states, impaired cognitive function, and impaired decision-making [5]. Athletes are particularly affected in the latter aspect.

As mentioned earlier, this sport is a game that requires constant decision-making, so if the duration and quality of sleep are not consistently prioritized, cognitive processes can be impaired, and consequently, performance results will be lower.

However, several studies demonstrate that sleep problems are common in professional athletes [9]. Furthermore, given that sleep is a critical and essential factor for performance, it is known that athletes experience interrupted sleep [10]. This evidence is reflected in another study [5] which asserts that women have better quality sleep than men in the same age range. Even research conducted by [10,11] states that during periods of high competition or in young athletes with dual careers, sleep problems can reduce attention span, increase the risk of errors, and hinder recovery.

Similarly, while it has been mentioned that sleep deprivation can negatively impact performance, it has also been proven to have negative consequences for a person’s health and well-being. Specifically, sleep deprivation for seven consecutive days was associated with a higher incidence of illness [10]. Their study demonstrated that the risk of illness was three times higher in those who slept less than seven hours. Another report observed that soccer players with shorter and poorer sleep duration showed a greater number and severity of injuries [6].

For these reasons, and taken together, these studies suggest that understanding injury risk in soccer players requires more than just physical or biomechanical indicators. It is also necessary to incorporate psychological variables such as those already mentioned: perceived stress, subjective recovery capacity, and its relationship to sleep habits. This approach fosters an integrative perspective that will allow for the future identification of at-risk athletes and the design of more effective preventative strategies that consider the athlete’s mental and emotional health. The aforementioned objective is to focus on the impact of sleep (quality, quantity, chronotype, etc.) on injury risk in soccer players, as well as to explore the role of related variables such as the stress–recovery balance.

## 2. Method

An exhaustive search was conducted using “Trobes UV,” a platform provided by the University of Valencia. Through this platform, studies were compiled from various databases, including ScienceDirect, PubMed, Ovid, EBSCO, MDPI, Springer Nature Link, SPORTDiscuss (with full text), and Dialnet.

The databases chosen for this study contained a large number of articles for searching for relevant information on the topic. To reduce this number, a keyword search was used: soccer, injury risk, football players, melatonin, sleep. The search term “sleep AND injury risk AND in football players” was also used. After searching for articles, the selection process was carried out. Both inclusion and exclusion criteria were established to facilitate the selection process. Table 1 which contains the inclusion–exclusion criteria shows the established selection criteria.

The “Exhibition of Interest” criterion refers to whether the participants in the studies must possess a particular characteristic to be included in this review. The “Type of athlete” criterion refers to whether the review should focus on a specific group of participants with similar characteristics. This differs from the “Participants” criterion, which is determined by the age group of the individuals in the articles. The context includes the location of the participants in the articles. The publication type for a systematic review is typically original research. Finally, the publication year of the selected articles is also considered.

Therefore, in each database before the screening of the studies, the number of articles was as follows:

− ScienceDirect: 111
− Pubmed: 222
− OVID: 84
− EBSCO: 15
− MDPI: 7
− Springer Nature Link: 110
− SPORTDiscuss with full text: 14
− Dialnet: 4

In addition to the articles found in the different databases, through the bibliographic search of many of them, three more studies could be added.

To carry out the screening process of all the records, it was necessary to first carefully examine the title, the keywords used, and the abstract to see if at first glance it was related to our topic.

Subsequently, he prepared to read the entire study in order to finally decide whether to use it or discard it.

**Figure 2:**
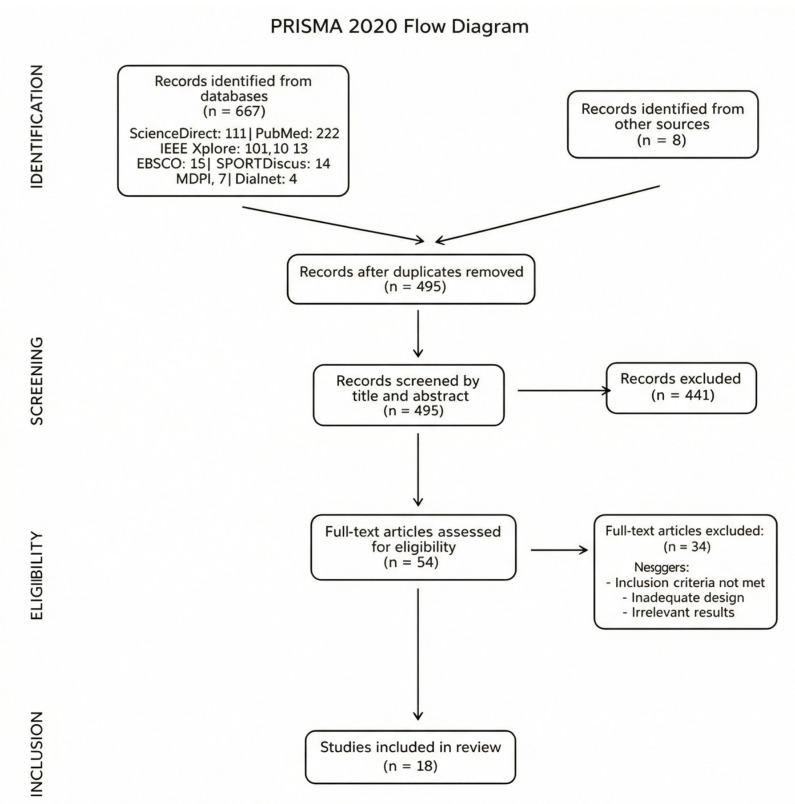
Flowchart.

Below, a flowchart is presented more graphically (Figure 2: flowchart), which explains the selection process.

This entire process described in the Methodology section was carried out following the guidelines of the PRISMA method [12].

This method involves following a set of recommendations to ensure that systematic reviews are as transparent and comprehensive as possible. This allows readers to understand how studies were identified, selected, and analyzed. The key components of a systematic review are as follows: Abstract, Introduction, Methodology, Results, Discussion, and the Flowchart (included in the Methodology section). In the Results section the reviewed studies were compiled in table format (Table 2).

Its applicability follows the same guidelines as those established in this section: establishing inclusion/exclusion criteria, identifying studies found in different databases, selecting articles that meet the established criteria, extracting the most relevant information from them, analyzing them by synthesizing the results and evaluating their quality, and finally, documenting the entire process in a flowchart.

Finally, to assess the methodological quality of the included studies, the Joanna Briggs Institute’s critical appraisal tools [13] were used, selecting the specific checklist for each type of study included in this systematic review: cross-sectional studies, cohort studies, case series, qualitative studies, and systematic reviews. For narrative reviews, the SANRA scale [14] was used. Each author independently reviewed and assessed each article, assigning a score based on compliance with the methodological criteria. Overall quality was classified as “High” (>75% compliance with the criteria), “Medium” (50–75%), or “Low” (<50%), considering the risk of bias inversely proportional to this quality (see Table 3).

**Table 2:** Study results.

References (Year)	Population and Age	Aim	Methodology	N = Art/Pers.	Sleep Variables (Quality/Quantity)	Risk of Injury/Illness	Stress–Recovery	Main Results
Vianna et al., 2024 [1]	Physiotherapists and female football players (18–30 years old)	Perceptions about injury prevention and practices in clubs	Qualitative study. (Interviews and surveys)	21 physiotherapists and 35 players	N/A	Perception of the importance of sleep for prevention	Recovery—including sleep—as a key factor in injury prevention.	Physiotherapists highlight the importance of sleep in preventing injuries
Molano Tobar and Molano Tobar, 2015 [2]	South American footballers (~20–35 years old)	Relationship between sociocultural and emotional factors and injuries	Qualitative study: Interviews, observation	~30 athletes and coaches	Sleep disorders as an emotional reflection	Increased risk linked to emotional exhaustion	Psychosocial suffering is a form of unmanaged stress.	Sleep disrupted by emotional and cultural burden
Kiziltoprak, 2020 [3]	Professional footballers (Age varies)	Analyze fatigue and recovery factors	Narrative review	N/A	Sleep = key to recovery/Insufficient sleep = fatigue	Fatigue = increased risk of injury	The imbalance between fatigue and recovery is the central axis of injury risk.	Good sleep = better recovery and prevention of fatigue
Laux et al., 2015 [4]	German professional footballers (~24 years old)	Analyze how the recovery– stress balance affects injury risk	Prospective study (1 season) (RESTQ-Sport, injury records)	22 players	Sleep included in recovery scale	High stress level = more injuries	It directly measures stress and recovery with RESTQ-Sport. The imbalance between the two predicts the risk of injury.	Good rest = less risk of injury
Chen, 2025 [5]	Professional players (19–34 years old)	Relationship between sleep quality and physical performance	Case study (PSQI, physical tests)	22 players	Related to performance/Less sleep = lower performance	Not evaluated	It does not include stress assessment tools.	Better sleep = better performance
Clemente et al., 2021 [6]	Professional footballers (Age varies)	Relationship between sleep, training load, and injuries	Systematic review	21 studies	Low quality/Insufficient	Increased risk with poor sleep	It analyzes how poor sleep interferes with player recovery and the ability to assimilate workloads.	Poor sleep = increased workload, fatigue, and injuries
Burke et al., 2020 [7]	College players (18–23 years old)	Association between sleep and sports injuries	Longitudinal (Actigraphy, records, questionnaires)	95 athletes	Worse in injured players/Shorter duration and efficiency	Higher incidence of injuries	Insufficient sleep impairs physiological recovery capacity.	Less sleep is linked to a higher risk of injury
Owoeye et al., 2024 [9]	University student-athletes (18–24 years old)	Relationship between sleep disorders and sports injuries	Cross-sectional observational study (PSQI, injury records)	203 athletes	58% with poor quality/Lower in injured	Significant association with injuries	A disruption of sleep due to academic and sporting stress.	Poorer sleep quality = more musculoskeletal injuries
Fitzgerald et al., 2019 [10]	Australian Rules football players (~18–30 years old)	Relationship between sleep, workload and disease incidence	Longitudinal cohort (Sleep, workload and infection records)	35 athletes	Worse sleep/<7 h = higher risk of illness	Relationship with respiratory infections	Indirect relationship: less sleep, greater perceived burden, and higher incidence of illness.	Less sleep, + high workload = more illness
Brett et al., 2021 [11]	Athletes with concussion (Young adults)	Evaluate the mediating role of sleep	Cross-sectional observational study (Sleep and depression questionnaires)	135 people	Decreased in shock/Shorter duration	Associated with depressive symptoms	Sleep as a mediator between previous shocks and depressive symptoms; emotional dimension of stress.	Sleep mediates between shock and depressive symptoms.
Bender et al., 2018 [15]	College and elite athletes (18–35 years old)	Validate sleep screening tool	Cross-sectional (ASSQ, clinical interviews)	199 athletes	Evaluated and Registered with ASSQ	Sleep disorders detected	The ASSQ can detect sleep patterns affected by stress.	ASSQ validated to identify sleep problems.
Biggins et al., 2019 [16]	Elite multi-sport athletes (18–35 years old)	Relationship between sleep and well-being	Cross-sectional PSQI, ASSQ, self-reported health	289 athletes	Altered PSQI/<6 h frequent	Poor sleep associated with poor health	Stress related to low mood and well-being; affects sleep and health.	Direct relationship between poor sleep and health
Bonnar et al., 2018 [17]	Athletes from different disciplines (Variable age)	Evaluate the effectiveness of interventions to improve sleep	Systematic review	17 studies	Variable/Improvement in most studies	Improved recovery	Revised interventions, such as relaxation, are aimed at reducing stress and improving recovery.	Effective interventions for rest and recovery.
Cook and Charest, 2023 [18]	Professional athletes (Variable Age)	Relationship between sleep, recovery, and performance	Narrative review	N/A	Poor performance in competition/Affects cognitive and physical functions	Increases fatigue and reduces recovery	It recognizes the impact of competitive stress on sleep quality.	Sleep is key to elite performance
Knufinke et al., 2018 [19]	Elite athletes from the Netherlands (18–35 years old)	Analyze sleep quality, quantity, and hygiene	Cross-sectional study (PSQI, sleep diary, SHI)	98 athletes	28% with poor quality (PSQI)/7.9 h + −1.1 h	Not directly evaluated	Poor sleep hygiene is associated with recovery difficulties.	Sleep hygiene influences reported quality
Krutsch et al., 2020 [20]	Amateur football players (~18–40 years old)	Analyze the relationship between training, sleep, and injuries	Prospective observational study (Questionnaire + injury records)	263 players	Worse in injured patients/Sleep <6 h in injured patients	Higher incidence of injuries with poor rest	Lack of recovery increases the likelihood of injury.	Sleep deprivation + poor preparation = more injuries
Silva et al., 2020 [21]	Brazilian professional footballers (18–30 years old)	Relationship between sleep quality and injury incidence	Prospective observational study (PSQI, medical records)	42 players	71% poor quality/Average 6.5 h	Direct relationship between poor quality and a higher number of injuries	No direct measurement of stress, but it acknowledges that poor sleep quality could reflect insufficient recovery.	Athletes with worse sleep had more muscle injuries
Stavrou et al., 2020 [22]	Athletes with sports injuries (20–35 years old)	Relationship between fitness indicators and sleep quality	Cross-sectional study (Sleep questionnaires, fitness tests)	102 athletes	Poor quality in injured/<7 h most	Injuries associated with poor sleep	There is a bidirectional relationship between fitness and sleep quality. Injured athletes experience greater physical and mental stress.	Poor sleep quality affects recovery and increases the risk of injury.

**Table 3:** Evaluation of methodological quality and risk of bias.

Reference	Study Design	Evaluation Tool	Criteria Met	Global Quality
Bender et al., 2018 [15]	Cross-cutting (Validation)	JBI Cross-Sectional	7/8	High
Biggins et al., 2019 [16]	Cross	JBI Cross-Sectional	7/8	High
Bonnar et al., 2018 [17]	Systematic Review	JBI Systematic Review	9/11	High
Brett et al., 2021 [11]	Cross	JBI Cross-Sectional	7/8	High
Burke et al., 2020 [7]	Longitudinal (Cohort)	JBI Cohort	9/11	High
Chen, 2025 [5]	Case Study (Series)	JBI Case Series	6/10	Medium
Clemente et al., 2021 [6]	Systematic Review	JBI Systematic Review	10/11	High
Cook and Charest, 2023 [18]	Systematic Review	SANRA Scale	4/6	Medium
Fitzgerald et al., 2019 [10]	Longitudinal (Cohort)	JBI Cohort	9/11	High
Kiziltoprak, 2020 [3]	Narrative Review	SANRA Scale	3/6	Medium
Knufinke et al., 2018 [19]	Cross	JBI Cross-Sectional	7/8	High
Krutsch et al., 2020 [20]	Prospective (Cohort)	JBI Cohort	10/11	High
Laux et al., 2015 [4]	Prospective (Cohort)	JBI Cohort	9/11	High
Molano Tobar, 2015 [2]	Qualitative	JBI Qualitative	7/10	Medium
Owoeye et al., 2024 [9]	Cross	JBI Cross-Sectional	6/8	Medium
Silva et al., 2020 [21]	Prospective (Cohort)	JBI Cohort	8/11	Medium
Stavrou et al., 2020 [22]	Cross	JBI Cross-Sectional	7/8	High
Vianna et al., 2024 [1]	Qualitative	JBI Qualitative	8/10	High

## 3. Results

As seen in the table summary, the studies encompass the different types of athletes, namely elite, semi-professional, and amateur; all of them registered with a sports federation and are participating in competitions. It was essential that the soccer players be under 40 years of age. This allowed for the observation of any differences that might exist between sleep factors and injuries throughout a player’s career.

Regarding the methodology of the articles, they encompass a wide variety, ranging from quantitative studies (cross-sectional, prospective, and age-matched cohort) and systematic reviews to qualitative studies. All of these approaches aim to observe the impact of three key sleep characteristics: the number of hours slept, the quality of sleep, and each footballer’s chronotype. Furthermore, they explore the potential relationship between these factors and injuries and/or illnesses.

In order to gather this information, most studies used a quantitative and qualitative methodology; instruments related to sleep were used, including PSQI (Pittsburgh Sleep Quality Index), ASSQ (Athlete Sleep Screening Questionnaire), actigraphy, chronotype questionnaires (MEQ, MCTQ) and interviews, sleep diaries, etc.

Additionally, an assessment of the methodological quality and risk of bias of the included studies was performed (see Table 3). After applying the JBI tools and the SANRA scale, it was found that most studies presented a high or medium methodological quality. Cross-sectional and cohort designs showed greater robustness due to the use of validated instruments, while case studies and narrative reviews presented a slightly higher risk of bias due to the nature of their designs. Nevertheless, all studies contributed data relevant to the objective of the review, and none were excluded based on quality criteria.

In their work, Vianna and cols. sought to establish physiotherapists’ perceptions of the causes of injuries in female soccer players. Through follow-up throughout the season, the physiotherapists reported that sleep is key in preventing illness and injuries [1].

It was a qualitative study of South American footballers where he reflected on the emotional and physical impact of sport, and in this case, football [2].

The systematic review of soccer players sought to establish the relationship between fatigue and recovery, and to identify the primary factor in improving recovery. Sleep factors (quality, quantity, and chronotype) were evaluated, and the study concluded that this strategy is key to promoting sleep. This is primarily because fewer hours of sleep are associated with a higher risk of injury [3].

A prospective study conducted over one season with German footballers analyzed the balance between how the risk of injury can be explained by recovery time and stress levels. It confirmed that sleep is a good predictor of injuries, and that obtaining adequate rest is a potential way to prevent them [4].

Through a case study of elite soccer players, he established the relationship between sleep and performance. His main findings showed that a better daily sleep routine was linked to better performance on the field [5].

In his review, he discusses training load, sleep, and injury risk. A study of professional soccer players revealed that most experienced poor sleep quality and insufficient sleep duration. The results showed that poor sleep quality, combined with insufficient sleep duration and a heavier training load, was associated with an increased risk of sports injuries [6].

Through his longitudinal study, he attempted to establish possible connections between sleep and injuries in college soccer players. He collected the information through self-reported measurements. He highlighted that individuals with injuries had poorer sleep quality and fewer hours of sleep. Therefore, the results showed that less sleep increased the risk of injury [7].

A study involving soccer and basketball players aimed to establish the relationship between sleep disorders and injuries. The results showed that sleep disturbances were associated with shorter and slower recovery times, and therefore, a higher number of injuries [9].

Fitzgerald and colleagues investigated Australian professional soccer. They conducted a longitudinal study with soccer players, demonstrating that increased training load and reduced sleep were stronger predictors of disease incidence [10].

Through this study of concussion, sleep, and depression, he wanted to establish the mediating role of sleep between these two injuries. The results confirmed that sleep quality acted as a mediator between concussion and depressive symptoms in athletes. Specifically, a person who suffered a concussion had fewer hours of sleep and of poorer quality [11].

Through Bender’s study, she conducted in-depth research on the reliability and validity of the Athlete Sleep Screening Questionnaire (ASSQ) for detecting sleep disturbances in athletes. Using this questionnaire, 25% of the participants did present clinical sleep problems, which required more intensive treatment [15].

With the aim of establishing a relationship between sleep and well-being, it was identified that 16% of the athletes presented significant sleep problems, associated with poor sleep hygiene (impaired sleep quality and <6 h slept) and poorer overall health. The data were collected through the PSQI, ASSQ, and self-reported health questionnaires in the study of [16].

In this study, he compiled numerous systematic reviews to address intervention plans for improving sleep and performance. He discovered that improving sleep hygiene (primarily increasing the number of hours of sleep), along with planned naps and cognitive–behavioral therapy, were among the best strategies for combating sleep problems [17].

Their narrative review synthesizes the evidence on sleep in athletes. Poor sleep quality affected numerous cognitive and physical functions. Furthermore, sleeping less than 7 h a day leads to greater fatigue and slower recovery, resulting in a higher risk of injury. This underscores the crucial role of sleep in athletic performance [18].

The sleep habits and hygiene of elite athletes were studied. For this reason, the quality and quantity of their sleep were analyzed in particular. Many reported that they were not receiving adequate rest due to the use of electronic screens before bed and their irregular daily schedules. Therefore, inadequate recovery would negatively impact their performance [19].

Unlike the other studies compiled, this work focuses on amateur soccer players. Specifically, it analyzed how a lack of preparation and sleep deprivation increased the risk of injury in soccer. It was confirmed that injured athletes slept poorly, averaging less than six hours. Therefore, sleep deprivation and poor physical preparation were associated with a higher injury rate [20].

In their study with Brazilian soccer players, they attempted to determine the incidence of injuries among those with poor sleep hygiene. The results confirmed his hypothesis. A total of 71% of the soccer players with poor sleep quality showed a higher incidence of injuries, establishing a direct link between sleep and injury [21].

It was concluded that injured athletes reported worse sleep, which implied a greater difficulty in the recovery period for footballers within the season schedule [22].

## 4. Discussion

After reviewing a wide variety of articles from different languages and time periods, which have been discussed previously, this study sought to establish the relationship and importance of sleep in its different dimensions, namely quality, quantity, and chronotype, while also considering stress and recovery as interrelated variables that influence both rest and risk in soccer players. Studies with different methodologies—qualitative and quantitative—published between 2015 and 2025 were included to observe the evolution of this factor over the last decade. Consequently, it has been shown that sleep is a determining factor in the prevention of sports injuries, as well as an optimizer of physical and cognitive performance.

It is understandable that elite footballers suffer numerous injuries throughout their careers and are therefore under greater scrutiny for injury prevention. However, for this reason, the inclusion criterion of amateur and professional footballers was used to differentiate between the two groups, in order to compare how sleep levels might affect performance in these categories.

Even at lower levels of competition (amateur football), the combination of sleep deprivation and poor physical preparation resulted in a higher-than-usual injury rate [20].

Furthermore, and simultaneously, a subtle comparison of gender perspectives was made, as the introduction already mentioned that female soccer players have a higher incidence of anterior cruciate ligament injuries. The physiotherapists emphasized that sleep is a central element in injury prevention in this population as well [1].

Both comparisons imply that sleep education should extend beyond professional sports, emphasizing that football is not just a man’s world.

After conducting this review, the findings of this study consistently show that footballers experience suboptimal sleep quality, characterized by insufficient duration, greater sleep fragmentation, and difficulty falling asleep. This confirms the significant link between sleep and injuries, making it one of the most important factors influencing well-being and recovery, regardless of whether individuals are athletes or not, and regardless of their level of competition. Insufficient sleep has negative effects on these vital aspects of a person’s life.

The vast majority of the dimensions addressed have been quantity, quality, and chronotype. All three have an effect on illness and injuries. However, some have a greater impact than others, always depending on external factors such as the demands of the championship, competitive pressure, and the clubs’ infrastructure. At the same time, intrinsic factors, such as the athletes’ level and physical condition, play a significant role.

On the one hand, poor sleep quality and insufficient rest hours were the two most frequently mentioned dimensions in the reviewed studies, which showed a strong association with a higher risk of sports injuries and illnesses [6,7]. Specifically, the risk of illness can be up to three times higher than usual [10].

On the other hand, chronotype (morning or evening preference) has been the least important dimension for this study, since there is less research on this term and it is more difficult to find evidence about it. Nevertheless, it is beginning to be considered essential within the field of sports.

The vast majority of athletes, in this case, soccer players, who have a longer afternoon training routine have greater difficulty adapting to morning training and competition schedules. However, morning training and competition could negatively affect performance and subsequent recovery, which, in turn, is associated with a higher probability of injury. Therefore, this dimension suggests the need to personalize training and competition schedules as much as possible, that is, to make them similar and aligned with each player’s circadian rhythm [16].

Compared to reviews prior to 2015, where sleep was considered a secondary factor, the studies analyzed show a significant advance in the multidimensional understanding of sleep. It has moved from being considered a simple rest process for the body and various cognitive functions to being understood as a biopsychosocial determinant of health and athletic performance.

Furthermore, from the perspective of sports psychology, the balance between stress and recovery has also proven to be a key factor in the injury predisposition of footballers, especially in most of the studies cited. Refs. [4,6,16] provide more precise evidence for these conclusions. The combined interaction of these psychological and physiological variables highlights the importance of adopting a biopsychosocial approach when studying the injury phenomenon.

On the other hand, some studies [11,17] emphasize that negative emotional aspects and states can mediate the effect of poor sleep on the athlete’s overall health.

In order to continue working on the topic of this work, it is true that small modifications must be made to obtain better results for future research, since there are still some relevant gaps.

Primarily, for a research paper to be as well-founded as possible, it should present longitudinal case studies spanning more than a certain period of time, and such studies remain scarce. This type of research allows for the establishment of clear causal relationships, and, to be more precise, more articles based on cases, at least from a regular season, should be published.

In addition to all this, there is scarce research on chronotype (already mentioned) in sports practice and the limited implementation of interventions (sleep hygiene, planned naps and the use of cognitive–behavioral techniques).

This gap represents an opportunity for future research, exploring how the combination of high training/match loads, poor sleep hygiene, and inadequate stress management cumulatively contribute to an increased risk of injury. The emergence and development of athlete-specific instruments such as the ASSQ (Athlete Sleep Screening Questionnaire) and the RESTQ-Sport (Recovery–Stress Questionnaire for athletes; Spanish version) has made it possible to record and perform screenings in sporting contexts, something unthinkable in past decades. However, there is still much studies to be conducted and much to explore further.

Furthermore, from a professional perspective, these findings reinforce the need to incorporate sleep assessment, monitoring, and management as an essential component of physical conditioning and injury prevention programs. Therefore, the use of validated tools, daily monitoring of sleep quality and duration, education on sleep hygiene, and strategic planning of training sessions (respecting circadian rhythms) are potential strategies that coaching staff should implement in the daily routines of footballers to maximize performance and protect their health.

### 4.1. Future Lines of Research

Finally, the review raises new questions that open up interesting avenues for future research. Some of these are as follows: How does chronotype influence injury risk and performance? What specific interventions can be applied in amateur football to improve sleep habits? Are there gender differences in the response to sleep deprivation in sports? And how can sleep education be integrated into football clubs and academies?

### 4.2. Limitations

However, the present study also has certain limitations that should be discussed. First, the time restriction applied to the article search was limited to the period 2015–2025; this allowed the analysis to focus on the most recent evidence, although it may have led to the exclusion of potentially relevant previous studies. Second, the selection of databases, although broad and diverse, did not include some key international repositories, such as Elsevier or Scopus, which could have reduced the comprehensiveness of the search strategy. Finally, the methodological heterogeneity observed in the studies prevented a meta-analysis, forcing the researchers to synthesize the findings in a narrative format, thus limiting the possibility of quantitatively estimating the overall effect of sleep on injury risk in soccer players.

## 5. Conclusions

In conclusion, sleep is emerging as a key and multidimensional factor in modern football, fundamental for competitive performance, physical recovery, and, importantly, injury prevention and the athlete’s mental health. Therefore, considering stress and recovery not as consequences of poor sleep, but as active modulating factors within the injury process, would allow for the design of more effective interventions focused on improving performance and promoting the footballer’s overall health; this is also considered one of the major challenges of applied sports science.

## Figures and Tables

**Table 1:** Inclusion-exclusion criteria.

Criteria	Inclusion	Exclusion
Exhibition of interest	People with and without injury. People with insomnia as a primary problem and without insomnia.	People with insomnia due to secondary causes: Medical disorders, psychiatric disorders, or substance abuse.
Type of athlete	Amateur, semi-professional and/or professional footballers	Youth footballers
Language	English, Spanish, Portuguese and Turkish	
Participants	Young people: 18–24 years old. Young adults: 25–40 years	Age group under 18 and over 40
Context	Professional and regional federated competitions	Non-federated competitions
Publication type	Systematic reviews, articles, case studies	Reviews, editorials, opinion pieces
Year of publication	2015–2025	Prior to 2015

## Data Availability

No new data were created or analyzed in this study.
